# Case Report: Successful surgical intervention for portal hypertension caused by primary hypoplasia of the portal vein in a dog

**DOI:** 10.3389/fvets.2025.1582290

**Published:** 2025-05-27

**Authors:** Joon-Ho Shin, Hyun-Jung Han

**Affiliations:** ^1^Department of Veterinary Emergency and Critical Care Medicine, College of Veterinary Medicine, Konkuk University, Seoul, Republic of Korea; ^2^KU Center for Animal Blood Medical Science, Konkuk University, Seoul, Republic of Korea

**Keywords:** portal hypertension, primary hypoplasia of the portal vein, splenectomy, ascites, dog

## Abstract

An 11-year-old, 5-kg castrated male Miniature Poodle presented with persistent ascites lasting 3 weeks. A thorough physical examination, comprehensive blood tests, and diagnostic imaging (including radiography, ultrasonography, and computed tomography) revealed ascites, splenomegaly, hepatomegaly, and decreased portal vein velocity (5–6.6 cm/s), leading to a diagnosis of portal hypertension. Prehepatic and posthepatic causes were ruled out, and the patient was diagnosed with intrahepatic portal hypertension. Despite conservative management with diuretics and a sodium-restricted diet, severe ascites persisted. A surgical liver biopsy via exploratory laparotomy was performed to determine the specific cause of intrahepatic portal hypertension. Concurrently, splenectomy was carried out after identifying splenomegaly and congestion, which were likely associated with portal hypertension. Histological examination of the spleen revealed mild-to-moderate congestion and complex nodular hyperplasia, while liver examination confirmed a diagnosis of primary hypoplasia of the portal vein (PHPV). Postoperatively, the dog experienced a transient increase in ascites and complications such as anorexia, mild anemia, and hypoalbuminemia, all of which were managed with supportive care. From postoperative day 4, the ascites completely resolved, and the portal vein velocity normalized (17–18 cm/s). Four months post-surgery, the patient showed no further signs of ascites. This case report describes the diagnosis and successful management of PHPV-induced portal hypertension in a dog, highlighting the efficacy of splenectomy in resolving ascites and improving portal vein hemodynamics in cases of PHPV in dogs.

## 1 Introduction

Portal hypertension (PH) is a pathological increase in pressure within the portal venous system. In human medicine, “PH” is defined by a hepatic venous pressure gradient (HVPG) greater than 6 mmHg, calculated as the difference between wedged hepatic venous pressure and free hepatic venous pressure (1). When this gradient exceeds 12 mmHg, complications such as variceal bleeding and ascites may occur (2). In veterinary medicine, direct or indirect measurement of portal venous pressure is rarely performed. Therefore, PH is typically diagnosed based on clinical evidence (e.g., ascites, multiple acquired portosystemic shunts, and hepatic encephalopathy) or portal vein hemodynamics assessed using Doppler ultrasound (2).

PH can be anatomically classified as prehepatic, intrahepatic, or posthepatic, with each type arising from elevated resistance in different parts of the portal venous system. Prehepatic PH is due to obstructions or extraluminal compression of the extrahepatic portal vein. Intrahepatic PH results from increased resistance within the microscopic portal vein tributaries, sinusoids, and small hepatic veins. Posthepatic PH is attributed to occlusion at the level of the larger hepatic veins, posthepatic caudal vena cava, or right atrium. Posthepatic causes are the most common contributors to portal hypertension in dogs and cats, with the most frequent examples being right-sided heart failure, pericardial disease, and pulmonary hypertension (2).

Primary hypoplasia of the portal vein (PHPV), a congenital vascular anomaly, is an uncommon cause of intrahepatic PH in dogs. In PHPV, the extrahepatic portal vein may be abnormally small or absent, and this can affect the intrahepatic vein in some cases (3). Noncirrhotic PH, microvascular dysplasia, idiopathic hepatic fibrosis, and hepatoportal fibrosis are terms that have been used to describe this condition. However, the World Small Animal Veterinary Association guidelines on canine and feline liver diseases have proposed standardizing the terminology for PHPV (2, 3). Clinical symptoms vary based on the severity of portal vein hypoplasia. In mild cases, PH may be absent, while in severe cases, symptoms of PH and hepatic encephalopathy—due to the development of multiple acquired portosystemic shunt (MAPSS)—may be observed (4).

Treatment for PHPV focuses on alleviating the symptoms of PH and preventing life-threatening complications. In dogs with PHPV-induced PH, medical management remains the primary approach, consisting of supportive care with a protein- and sodium-restricted diet, diuretics (e.g., furosemide and spironolactone), and antiulcer agents. Dogs with MAPSS secondary to PH are managed with a low-protein diet, antibiotics, and lactulose (5, 6). To date, however, there have been no published reports on the surgical treatment of PHPV in dogs.

This report presents a case of PHPV-induced PH in a dog, where refractory ascites was successfully managed with splenectomy. To the best of our knowledge, this is the first published report on the surgical treatment of PH caused by PHPV in dogs.

## 2 Case description

**Figure 1:**
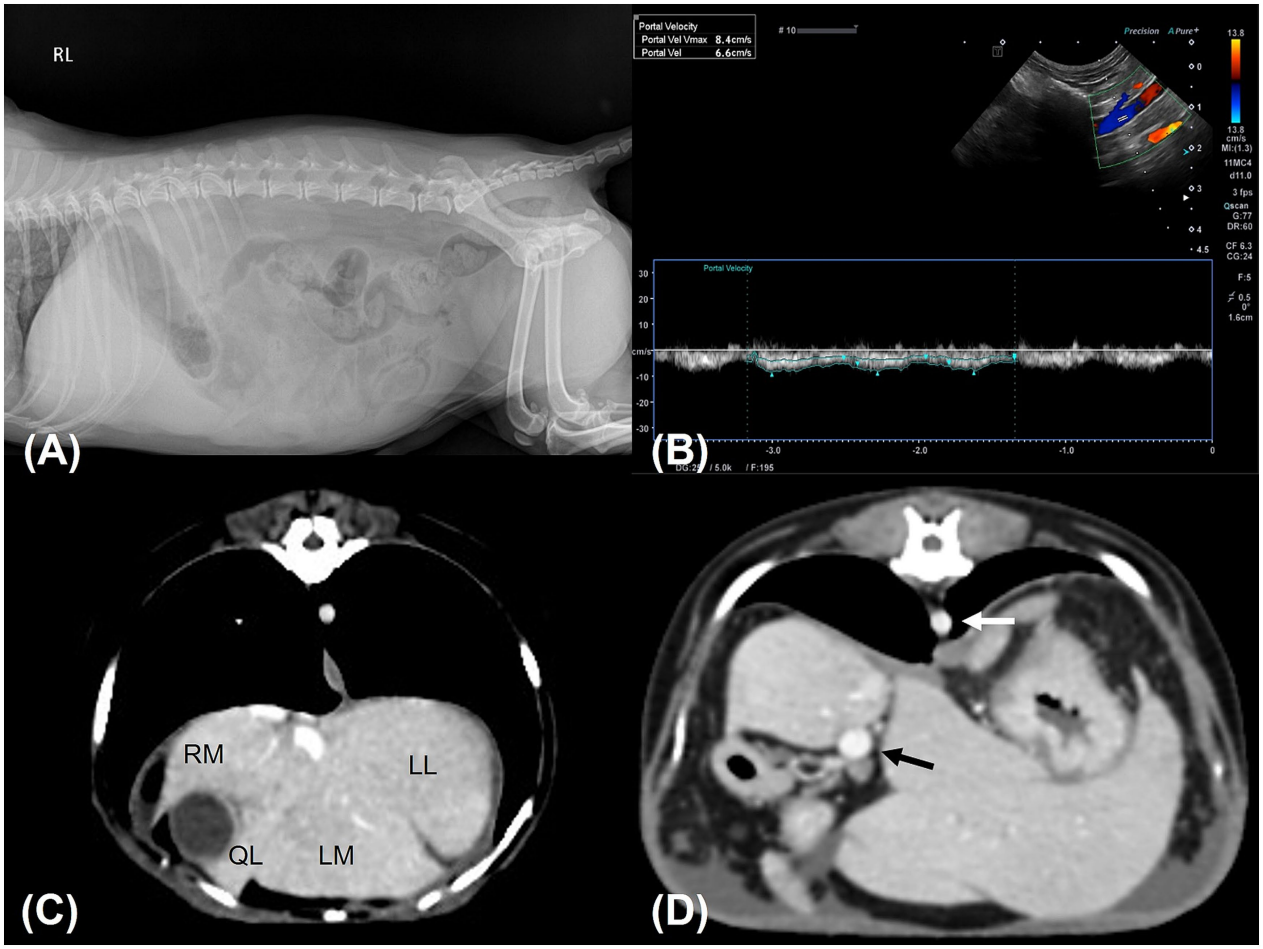
Diagnostic images. **(A)** Right lateral abdominal radiograph shows hepatomegaly and suggests the presence of ascites. The liver had rounded, blunt margins and extended well beyond the costal arch. Gastric axis displacement and decreased serosal detail were observed. **(B)** Abdominal Doppler ultrasound examination of the portal vein revealed decreased portal vein velocity (6.6 cm/s). **(C,D)** transverse CT scanning images. **(C)** The right hepatic lobes were assessed to be smaller in volume than the left hepatic lobes. Some interlobar spaces appeared to be delineated by ascites. **(D)** The ratio of the diameter of the portal vein (black arrow) to that of the aorta (white arrow) was 1.1, indicating a portal vein of normal size. RM; right medial lobe, LL; left lateral lobe, LM; left medial lobe, QL: quadrate lobe.

An 11-year-old, castrated male Miniature Poodle weighing 5 kg was referred to the Konkuk University Veterinary Medical Teaching Hospital for the evaluation of persistent ascites lasting 3 weeks. The dog had no known history of chronic disease, prior abdominal surgery, or hepatic dysfunction. According to the referring veterinarian and the owner, the patient had been in good health prior to the onset of abdominal distension. The patient had been prescribed furosemide (0.5 mg/kg BID) and spironolactone (1 mg/kg BID) by a local veterinary clinic for 7 days. Abdominocentesis was performed twice, 1 week apart, with 600 mL of fluid removed during the first procedure and 400 mL during the second. At presentation, the dog was bright, alert, and responsive. Physical examination revealed a body condition score of 5/9, abdominal distension without pain on palpation, mild hyperthermia (39.5°C), a normal pulse rate (155 beats/min) with a mild left systolic murmur, a systolic blood pressure of 150 mmHg (measured via Doppler), and tachypnea (60 breaths/min). Hematology, electrolyte analysis, and serum biochemistry were unremarkable. Hepatobiliary enzyme levels, including aspartate aminotransferase (AST), alanine aminotransferase (ALT), alkaline phosphatase (ALP), and gamma-glutamyl transferase (GGT), along with liver function markers such as total bilirubin, blood urea nitrogen, total cholesterol, glucose, and albumin, were all within reference ranges. Coagulation tests, including thromboelastography, prothrombin time (PT) and activated partial thromboplastin time (aPTT), were also unremarkable, except for an elevated D-dimer concentration (1002.78 ng/mL; reference range, 50–250 ng/mL). Diagnostic imaging, including abdominal radiography and ultrasonography, confirmed the presence of abundant ascites. Analysis of the abdominal fluid identified it as non-septic modified transudate (total nucleated cell count: 0.96 k/μL, total protein: 3.5 g/dL). Abdominal radiography revealed hepatomegaly (Figure 1A), whereas ultrasonography showed infiltrative/vacuolar hepatopathy, irregular liver margins, splenomegaly, and decreased portal vein velocity (5–6.6 cm/s) (Figure 1B). Echocardiography indicated mild mitral regurgitation and stage B1 myxomatous mitral valve disease (American College of Veterinary Internal Medicine), with a left atrium-to-aorta ratio of 1.37 and a left ventricular end-diastolic internal diameter normalized to body weight of 1.47. No abnormalities in the right heart were observed. Computed tomography (CT) revealed microhepatica in the right medial and lateral liver lobes, hepatomegaly in the remaining lobes, and splenomegaly (Figure 1C). Additionally, no evidence of portal vein thrombosis or shunting to the caudal vena cava was noted. The portal vein size and pulmonary vein-to-aorta ratio (1.1) were within reference ranges (Figure 1D). Based on the findings of ascites, splenomegaly, and reduced portal vein velocity, the patient was diagnosed with PH. Prehepatic and posthepatic causes of PH were excluded via diagnostic imaging, confirming intrahepatic PH.

On presentation day, 410 mL of ascitic fluid was removed, and the dog’s weight was 4.4 kg. Management of ascites included furosemide (0.75 mg/kg BID), spironolactone (1 mg/kg BID), and a sodium-restricted diet for 4 days. However, despite these interventions, 400 mL of ascites was removed on the fourth day after presentation. Portal vein velocity remained low at 4.5 cm/s, and D-dimer levels increased to 2000 ng/mL (reference range: 50–250 ng/mL). Furosemide dose was increased to 1 mg/kg BID and spironolactone to 1.5 mg/kg BID for 19 days. Nonetheless, 350 mL of ascites was removed on the 10th and 18th days post-presentation. To definitively determine the cause of the PH, additional diagnostic tests, including liver biopsy, bacterial and fungal cultures of liver tissue, and liver panel tests (including canine adenovirus 1 and 2 polymerase chain reaction (PCR) and measurement of copper concentration), were planned. Considering the risk of thrombosis due to splenomegaly and its potential impact on portal vein hemodynamics, splenectomy was also considered.

On the 23rd day post-presentation, laparotomy was performed for liver biopsy and examination of the spleen and other abdominal organs. Premedications included cefazolin [30 mg/kg, intravenous (IV)], famotidine (1 mg/kg, IV), maropitant (1 mg/kg, IV), butorphanol (0.1 mg/kg, IV), and midazolam (0.3 mg/kg, IV). Anesthesia was induced with propofol (6 mg/kg, IV), the patient was subsequently intubated, and anesthesia was maintained with isoflurane (1.5–2.0%) in oxygen (0.25–0.5 L/min). Following aseptic preparation, routine ventral midline celiotomy was performed.

**Figure 2:**
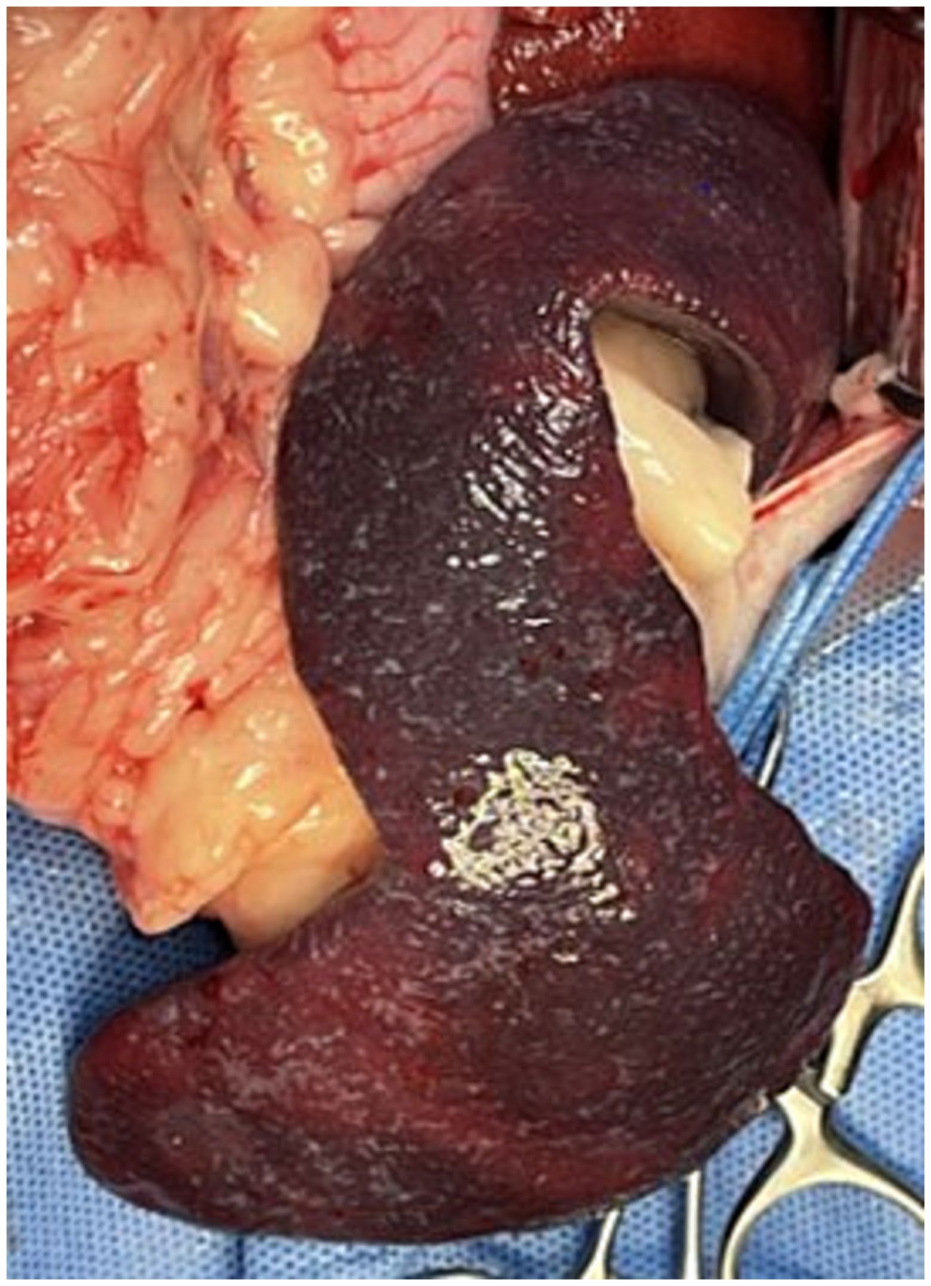
Macroscopic images during surgery. The spleen was markedly enlarged with irregular borders and exhibited a diffuse dark red discoloration, indicative of congestion.

Upon entering the abdominal cavity, approximately 400 mL of light-yellow, transparent peritoneal fluid was removed using constant suction. Exploratory laparotomy revealed an enlarged spleen with irregular margins and signs of congestion (Figure 2). The liver appeared irregular with small right lateral and medial lobes, while the remaining lobes were hypertrophic. The other organs exhibited no remarkable findings on gross examination. A liver tissue sample was obtained from each lobe using the guillotine technique, and two additional samples were obtained from the left lateral lobe using a punch biopsy. Hemostasis was achieved at the punch biopsy sites using hemostatic sponges (Cutanplast, Mascia Brunelli, Milan, Italy). The enlarged and congested spleen was resected using a LigaSure vessel-sealing device (Covidien Inc., Mansfield, MA, United States). The abdominal cavity was copiously lavaged with warm sterile saline solution (0.9% NaCl) and routinely closed. The sampled liver tissue was subjected to histopathology, bacterial and fungal cultures, canine adenovirus 1 and 2 PCR, and copper concentration analysis.

**Figure 3:**
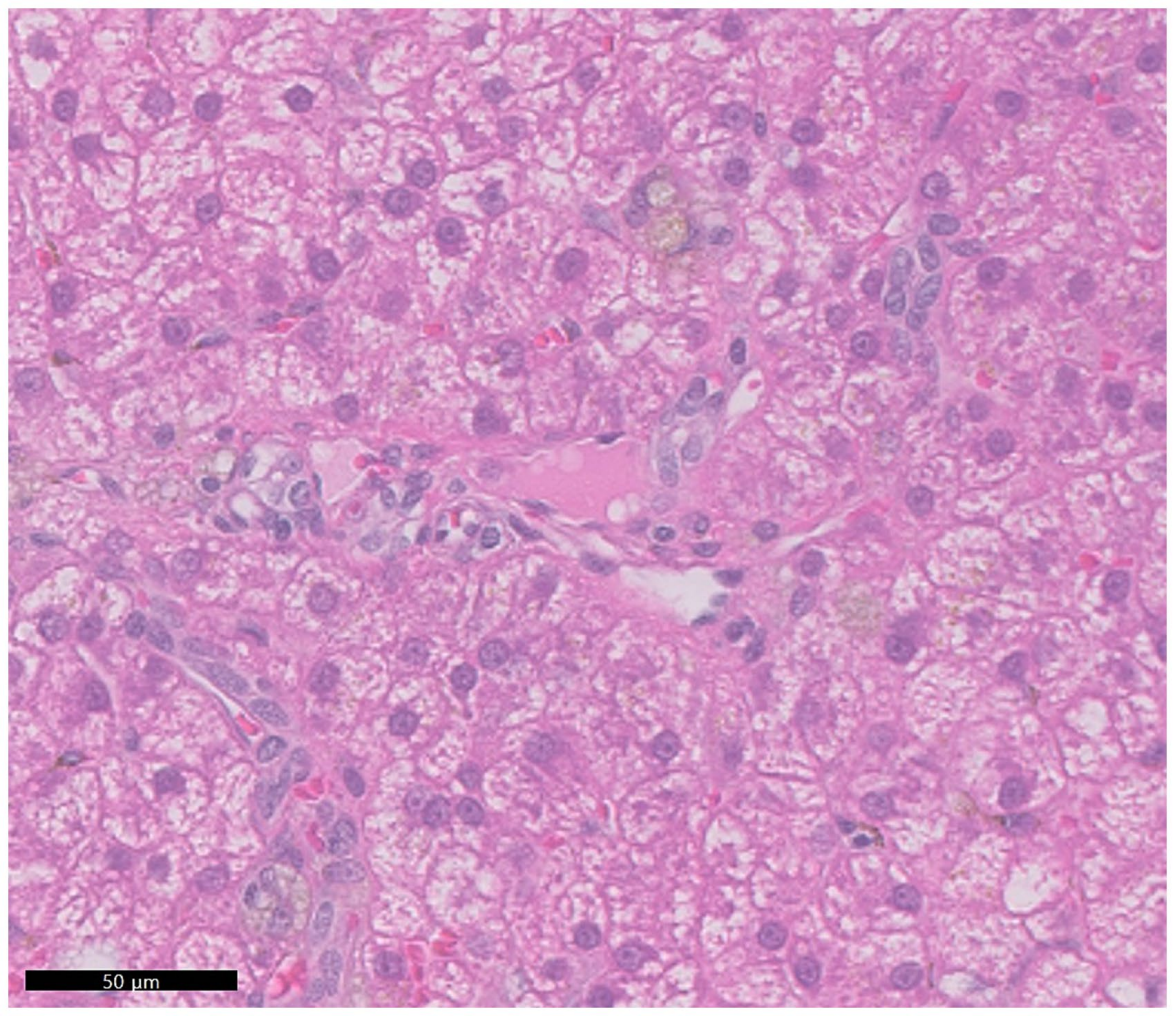
Representative histopathologic features observed across all hepatic lobes. The portal veins were small and hardly discernible. A diffuse moderate vacuolar degeneration was noted within the cytoplasm of hepatocytes. Hepatocytes often exhibited mild accumulations of pale brown granular pigment, possibly indicating deposition of lipofuscin or iron.

Histological examination of the liver revealed inconsistent portal vein hypoplasia, multifocal aberrant microvasculature, moderate-to-marked vacuolar degeneration, mild-to-moderate intracytoplasmic hepatocellular pigment accumulation, and multiple lipogranulomas (Figure 3). These findings were consistent across all liver lobes, with no histological differences between the right lobes with microhepatica and the enlarged left lobes. The changes were associated with PHPV. The copper concentration in liver tissue was within the normal range (152 ppm, dry weight). PCR tests for canine adenovirus types 1 and 2, which cause infectious canine hepatitis (ICH), were negative, and no bacteria or fungi were detected in the liver tissue culture. Histological examination of the spleen revealed mild-to-moderate congestion and complex nodular hyperplasia. Based on histological findings, diagnostic imaging, and laboratory tests, the patient was diagnosed with PHPV.

**Figure 4:**
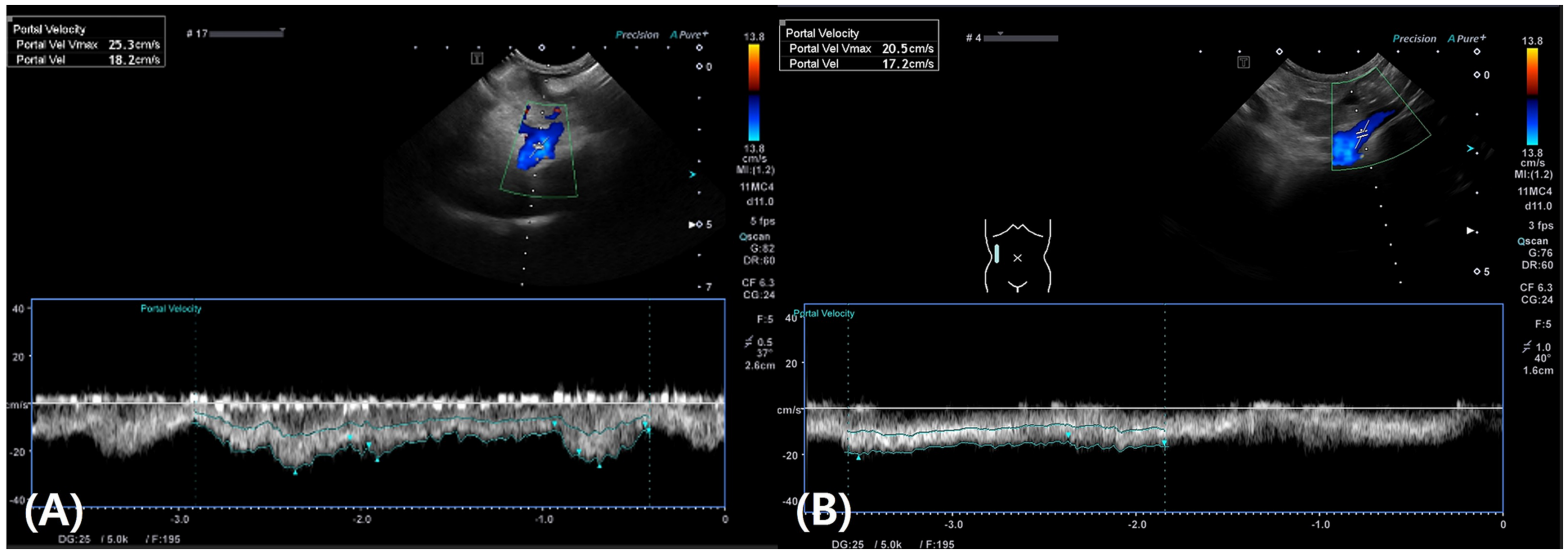
Abdominal Doppler ultrasound images. **(A)** On postoperative day 4, the portal vein velocity returned to the normal range (18 cm/s). **(B)** On postoperative day 10, the portal vein velocity remained within the normal range at 17 cm/s.

On postoperative day 1, the patient exhibited vomiting, anorexia, lethargy, abdominal distension, and hypotension (systolic blood pressure of 80 mmHg, measured by Doppler). Blood tests revealed mild anemia (hematocrit, 31.3%; reference range: 37.3–61.7%), leukocytosis (white blood cell count, 33.3 K/μL; reference range: 5.05–16.76 K/μL), hypoalbuminemia (albumin, 1.9 g/dL; reference range: 2.2–3.9 g/dL), elevated C-reactive protein (>10 mg/dL; reference range: 0.1–1 mg/dL), and elevated liver enzymes, including AST (131 U/L; reference range: 0–50 U/L) and ALT (192 U/L; reference range: 10–125 U/L). Additionally, canine pancreas-specific lipase (830 ng/mL; reference range: 200–400 ng/mL) and D-dimer levels (2064 ng/mL; reference range: 50–250 ng/mL) were elevated. Postoperative treatment included a continuous infusion of fentanyl (4 μg/kg/h) for the first 12 h, followed by fluid therapy, fresh frozen plasma transfusion (10 mL/kg IV BID) for 5 days, maropitant (1 mg/kg IV once daily), ondansetron (1 mg/kg IV BID), and continuous infusion of metoclopramide (1.5 mg/kg/day IV) for 7 days, and continuous infusion of famotidine (8 mg/kg/day IV) for 3 days. On postoperative day 1, 349 mL of ascitic fluid was drained, and 350 mL was removed on day 3. Both samples were classified as modified transudates. By postoperative day 4, the patient showed improvement, with resolution of vomiting and restoration of appetite. Abdominal ultrasound revealed a portal vein velocity of 18 cm/s, which was within the reference range (Figure 4A). With no further increase in abdominal effusion since the last drainage on postoperative day 3, the patient was discharged on postoperative day 7 after confirmation of improvements in blood tests and clinical symptoms.

On postoperative day 10, abdominal ultrasound revealed a portal vein velocity of 17 cm/s, still within the reference range, with no further effusion detected (Figure 4B). Liver lesions (diffuse hyperechogenicity and irregular margins) remained unchanged except in the biopsy area. At the final follow-up on postoperative day 15, the patient showed good vitality, normal appetite, no abdominal distension, and a stable weight of 4.2 kg. Complete blood count, serum biochemistry, including pre- and postprandial total bile acid concentrations, and coagulation tests were all within reference ranges.

Long-term follow-up information was obtained from the owners via telephone 4 months after surgery. The owner reported that the patient no longer experienced abdominal distension due to ascites and that the patient’s body weight remained consistent.

## 3 Discussion

PHPV typically presents with clinical signs in dogs under 2 years of age and is reported to be more common in breeds such as Yorkshire Terriers and Toy Poodles (2, 4, 7). In mild cases, PH may be absent; however, persistent or intermittent slight elevations in liver enzymes, such as ALT, ALP, AST, and GGT, can occur. In more severe cases, clinical manifestations include neurological signs, polyuria, polydipsia, intermittent gastrointestinal symptoms, lethargy, and ascites (4). In this case, the dog presented with ascites related to PH caused by PHPV at the advanced age of 11 years. Although PHPV is a congenital vascular anomaly, it is presumed to be a progressive condition based on reports of PHPV symptoms manifesting in older dogs and documentation of elevated serum levels of total bile acids over time in dogs with PHPV (4). In this case, ascites—a clinical manifestation of PH—was observed without any elevation in liver enzyme levels, indicating that severe clinical signs of PHPV may not necessarily be accompanied by elevated liver enzyme levels.

The diagnosis of PHPV requires a liver biopsy along with imaging studies to rule out other conditions such as portosystemic shunts, arteriovenous fistulas, or portal vein obstruction (4, 8). The histological features of PHPV include diminished portal vasculature and histopathological evidence of hepatic hypoperfusion in the absence of inflammation and nodular regeneration. Depending on the degree of portal vein hypoplasia, compensatory arteriolar hyperplasia and hepatocellular atrophy may occur. These are often accompanied by vacuolar degeneration, portal tract fibrosis, bile duct proliferation, lipogranuloma formation, and sinusoidal dilation (2, 9–11). In this case, imaging studies, including ultrasonography and CT, as well as liver biopsy, culture tests, PCR tests, copper concentration analysis, and exclusion of obstructive conditions (e.g., Budd-Chiari syndrome and thrombosis) and liver diseases (e.g., liver cirrhosis, fibrosis, hepatitis, copper storage hepatopathy, and infection), were performed. Liver biopsy revealed inconsistent portal vein hypoplasia and evidence of compensatory changes or hepatic hypoperfusion associated with PHPV, including multifocal aberrant microvasculature, moderate-to-marked vacuolar degeneration, mild-to-moderate intracytoplasmic hepatocellular pigment accumulation, and multiple lipogranulomas (2, 9–11). Based on diagnostic imaging and histopathological results, PHPV was diagnosed as the cause of PH and ascites.

In this case, PH was identified using Doppler ultrasound, which revealed a low portal vein velocity. Ascites and splenomegaly were considered complications of PH. The mechanism by which PH induces ascites involves changes in the systemic and splanchnic circulation. In these circulations, relatively increased production of nitric oxide (NO) leads to a decrease in effective circulating volume due to reduced systemic vascular resistance. This stimulates arterial baroreceptors, activates the sympathetic nervous system, and increases the secretion of the renin-angiotensin-aldosterone system and antidiuretic hormones. These responses result in hyperdynamic circulation and salt and water retention, leading to ascites (1).

Currently, there is no specific treatment for PHPV-induced PH in veterinary medicine. In dogs, the primary approach involves managing ascites through sodium restriction and diuretics (6). In humans, treatment for idiopathic noncirrhotic portal hypertension (INCPH), similar to PHPV-induced PH in dogs, primarily focuses on managing variceal bleeding, which is the most critical complication when the underlying cause of PH cannot be treated. This is achieved using nonselective beta-blockers or endoscopic variceal ligation (12). In refractory cases, shunt interventions or liver transplantation may be used (12, 13). Shunt interventions are classified as selective or non-selective. Selective shunts, such as the distal splenorenal shunt and small-diameter transjugular intrahepatic portosystemic shunt (TIPS), divert partial portal flow, preserving hepatic function and reducing the risk of hepatic encephalopathy. Non-selective shunts, including the portocaval shunt and large-diameter TIPS, divert total portal flow, offering better variceal bleeding control but with a higher risk of encephalopathy (14). In dogs, treatments other than sodium restriction and diuretics have not been reported, and most cases have resulted in euthanasia (6).

In humans, surgical treatments such as splenectomy and partial splenic embolization are used to manage INCPH complications such as variceal bleeding, splenomegaly, and hypersplenism (15). These procedures reduce portal venous flow, thereby decreasing PH (15). However, there have been no attempts at surgical treatment, such as splenectomy, for PHPV-induced PH in veterinary medicine. In this case, despite the absence of variceal bleeding, refractory ascites persisted despite supportive care. Based on the mechanism of splenectomy in humans, splenectomy was performed, resulting in complete resolution of ascites by postoperative day 3 and normalization of portal vein velocity (16).

We believe that splenectomy led to the normalization of portal vein velocity, and the resolution of ascites was as follows: First, splenectomy reduces portal venous inflow by eliminating congested blood flow from the spleen (17). Second, splenectomy may decrease the level of endothelin-1 (ET-1) and increase the level of NO in hepatic venous blood. ET-1, a vasoconstrictor, regulates hepatic-stellate cell (HSC) contractility and modulates intrahepatic portal vascular resistance (1, 18). Removing spleen-derived ET-1 relaxes stellate cells, thereby reducing intrahepatic portal vascular resistance. Improved sinusoidal circulation increases shear stress on sinusoidal endothelial cells, restoring NO production. Third, splenectomy may reduce both ET-1 and NO levels in peripheral blood. The elimination of spleen-derived ET-1 decreases endothelial NO production mediated by endothelin receptor B. Although the increase in NO concentration in hepatic venous blood and the decrease in NO concentration in peripheral blood may appear contradictory, these findings suggest that splenectomy has the potential to improve intrahepatic portal vascular resistance, as well as splanchnic and systemic hyperdynamic circulation (18). Consequently, despite not addressing the underlying hepatic vascular abnormalities, such as portal vein hypoplasia or aberrant microvasculature, splenectomy still resulted in the normalization of portal vein velocity.

This case report had some limitations. Portal vein pressure was neither directly nor indirectly measured during the diagnosis of PH. Direct measurement can be achieved via catheterization of the portal vein or its tributaries using a manometer (cmH2O) or pressure transducer (mmHg) (2). Indirect measurement involves inserting a balloon-tipped catheter into the hepatic vein and measuring both free and wedged hepatic vein pressures (2). However, due to the invasive nature of these procedures, portal hypertension in this case was diagnosed based on decreased portal vein blood flow velocity observed on Doppler ultrasound. For long-term evaluation, although follow-up assessments such as clinical examinations, blood tests, ultrasonography, and CT scans would have been ideal, only a brief phone call with the owner was possible. The owner declined further testing due to the patient’s stable condition without recurrence of symptoms, financial constraints, and the long distance from the hospital, which posed an additional limitation. Moreover, given the limitations of a single case report, definitive conclusions about the efficacy of splenectomy for PHPV-induced PH cannot be drawn. Further research and case studies are needed to elucidate the specific effects of splenectomy in PHPV-induced PH and establish clear criteria for its use based on disease severity. Nonetheless, the preliminary findings suggest that splenectomy may be a viable management strategy for this condition.

## 4 Conclusion

This case report describes the diagnosis and successful management of PHPV-induced PH in a dog, highlighting the efficacy of splenectomy in resolving ascites and improving portal vein hemodynamics. Splenectomy may represent a potential therapeutic option for managing ascites in dogs with PHPV-induced PH that do not respond to medical treatment.

## Data Availability

The original contributions presented in the study are included in the article/supplementary material, further inquiries can be directed to the corresponding author.
